# Supplementary figures and images for: Validation of artificial intelligence-based digital microscopy for automated detection of Schistosoma haematobium eggs in urine in Gabon
Source: PLoS Negl Trop Dis. 2024 Feb 23;18(2):e0011967. doi: 10.1371/journal.pntd.0011967 (PMC10917302; doi:10.1371/journal.pntd.0011967)

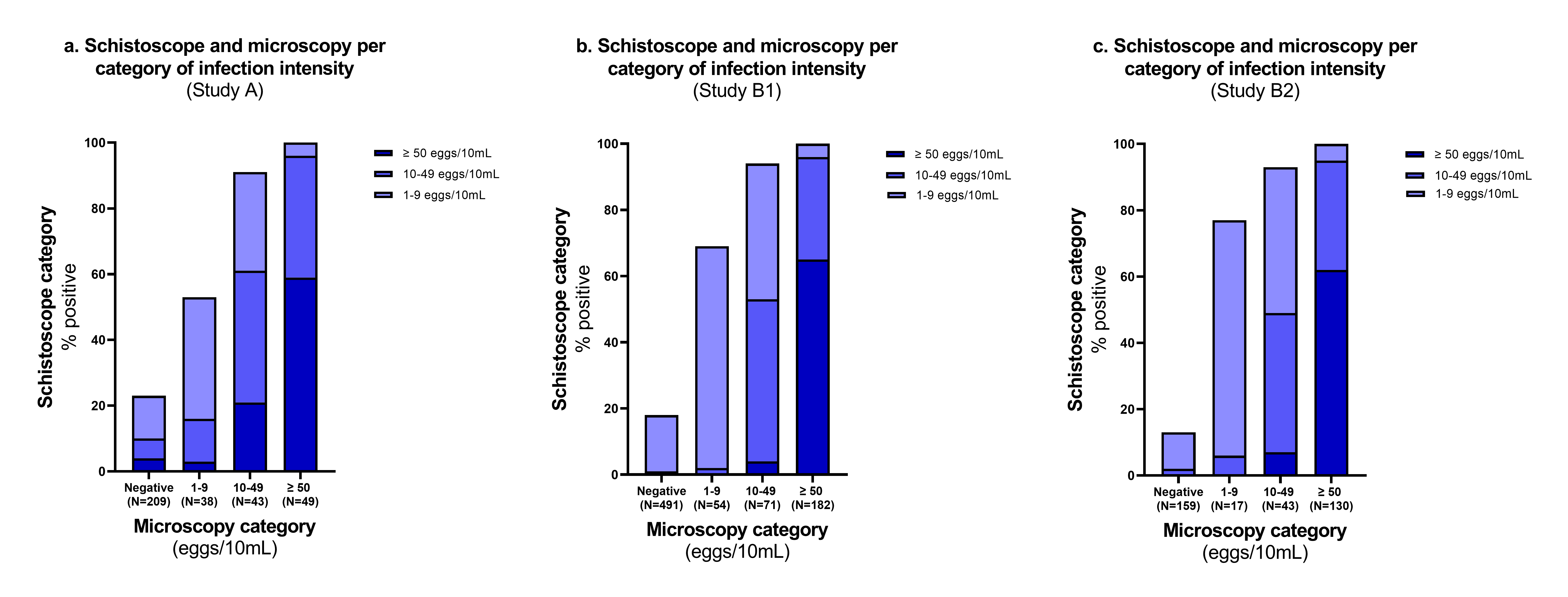

Supplement: S1 Fig — Agreement between the Schistoscope and microscopy per category of infection intensity in study A and B. (TIF) [file pntd.0011967.s002.tif]

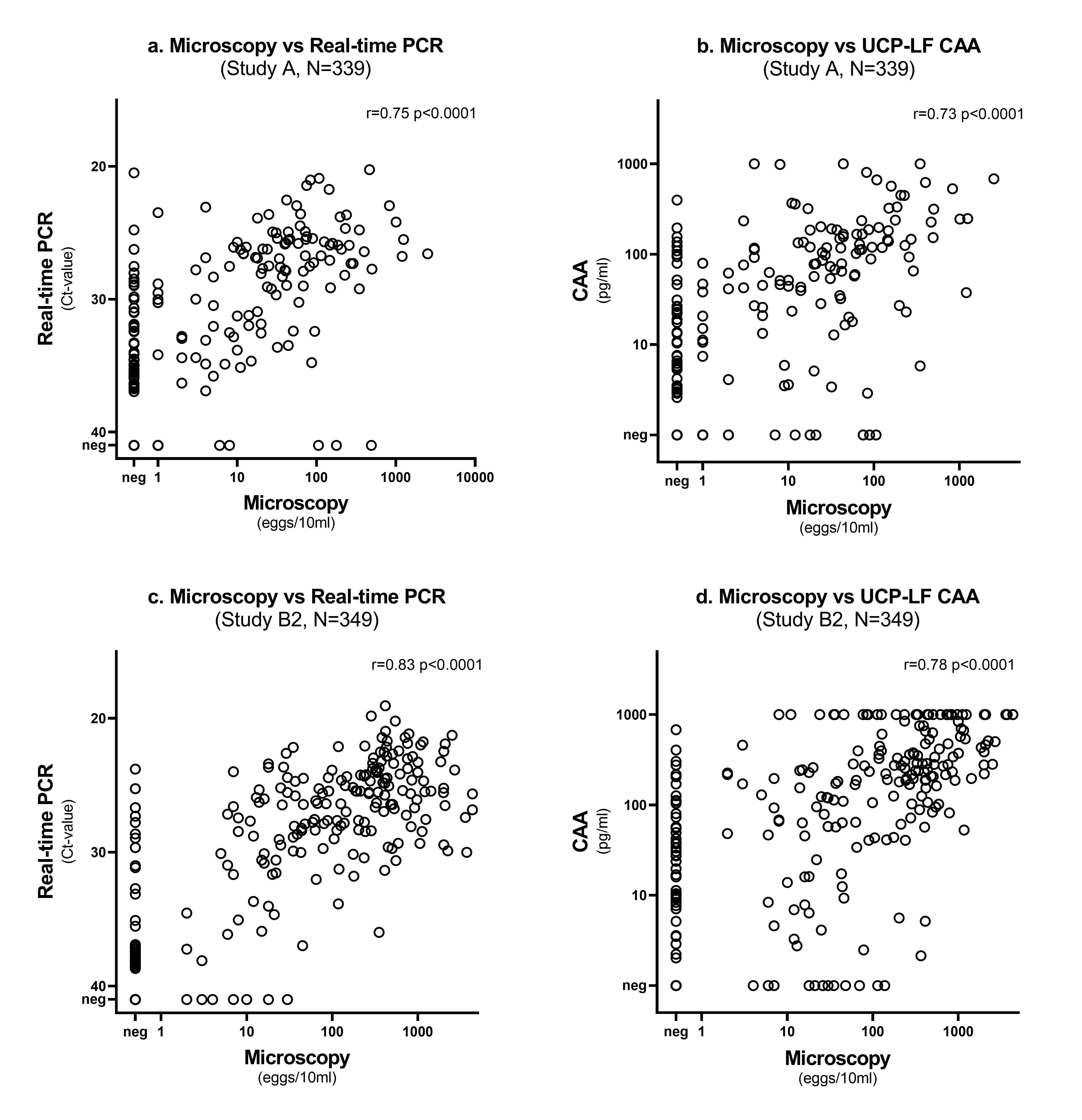

Supplement: S2 Fig — Correlation between S. haematobium egg counts measured by the conventional microscopy and Ct-values determined by by real-time PCR (a, c) and urine CAA concentration. (TIF) [file pntd.0011967.s003.tif]

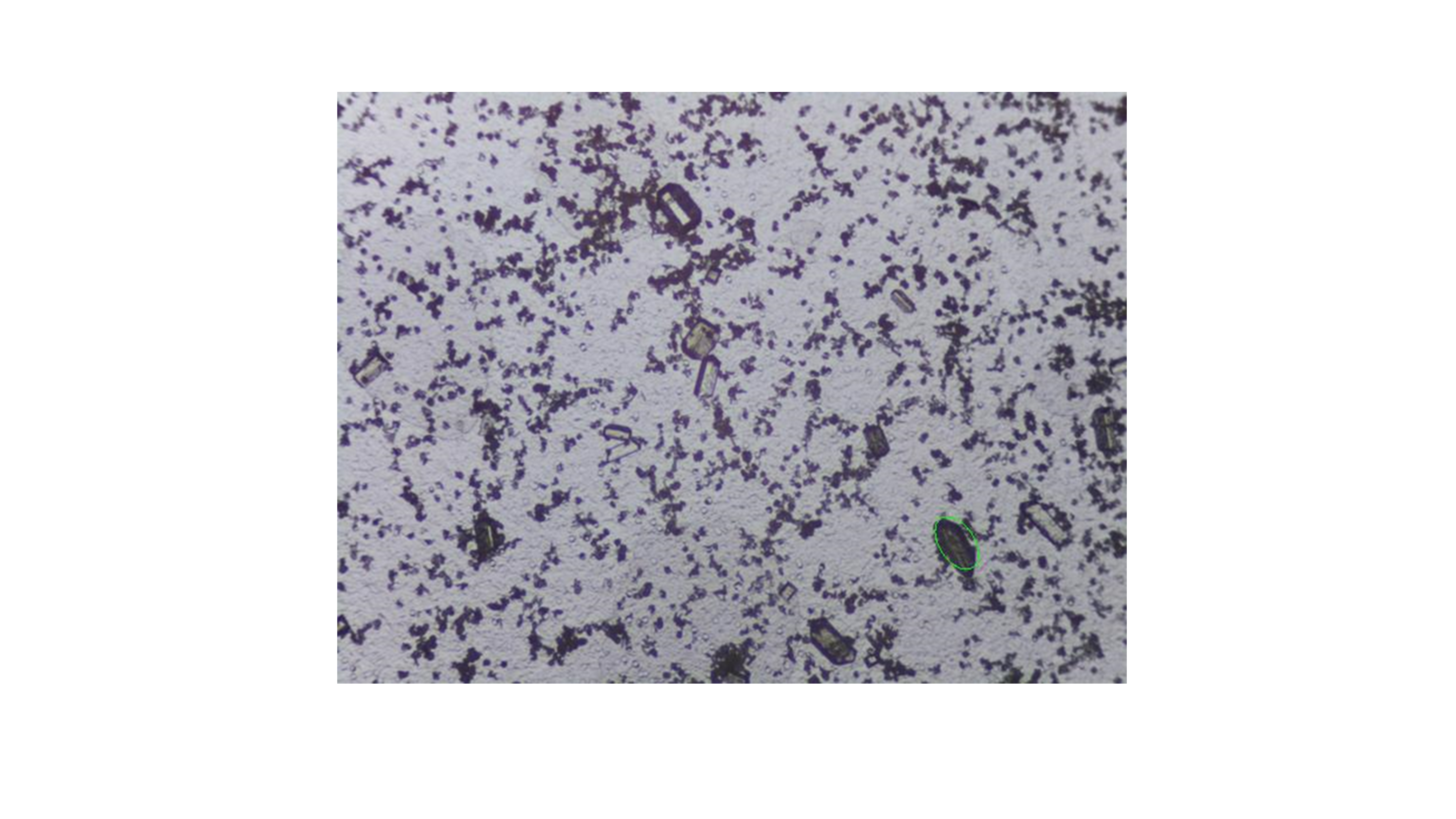

Supplement: S3 Fig — (TIF) [file pntd.0011967.s004.tif]
