# Supplementary material for: Validation of artificial intelligence-based digital microscopy for automated detection of Schistosoma haematobium eggs in urine in Gabon
Source: PLoS Negl Trop Dis. 2024 Feb 23;18(2):e0011967. doi: 10.1371/journal.pntd.0011967 (PMC10917302; doi:10.1371/journal.pntd.0011967)
Supplement: S1 Manual — (PDF) [file pntd.0011967.s005.pdf]

SCHISTOSCOPE 5.0

USER MANUAL

INclusive diagno**S**tics for **P**overty **RE**lated parasitic **D**iseases (INSPIRED)

The Schistoscope

Front View

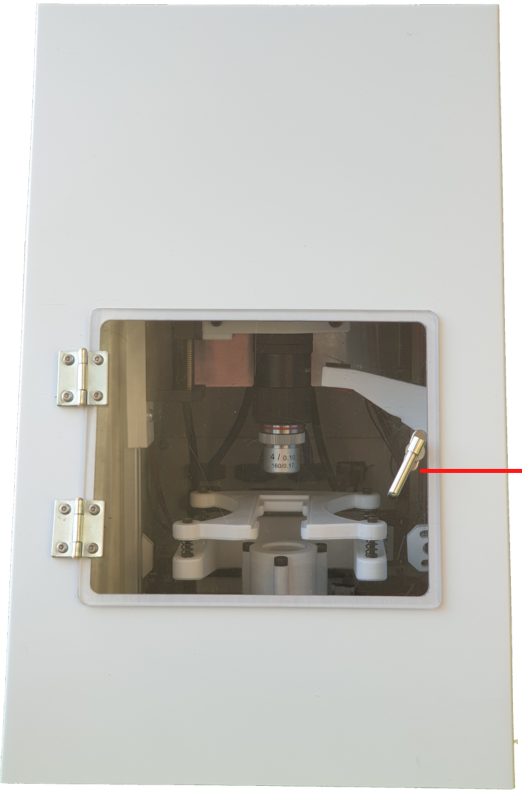

Back View

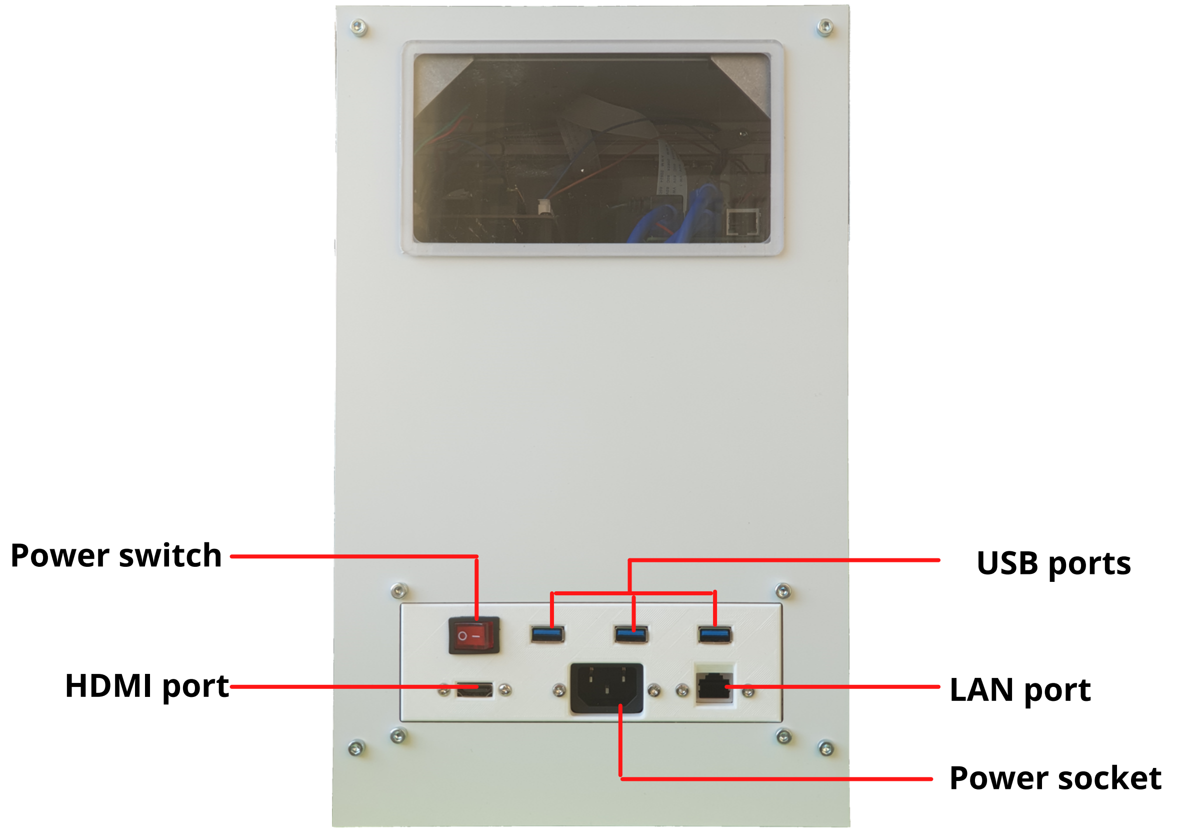

Controls

Graphical User Interface

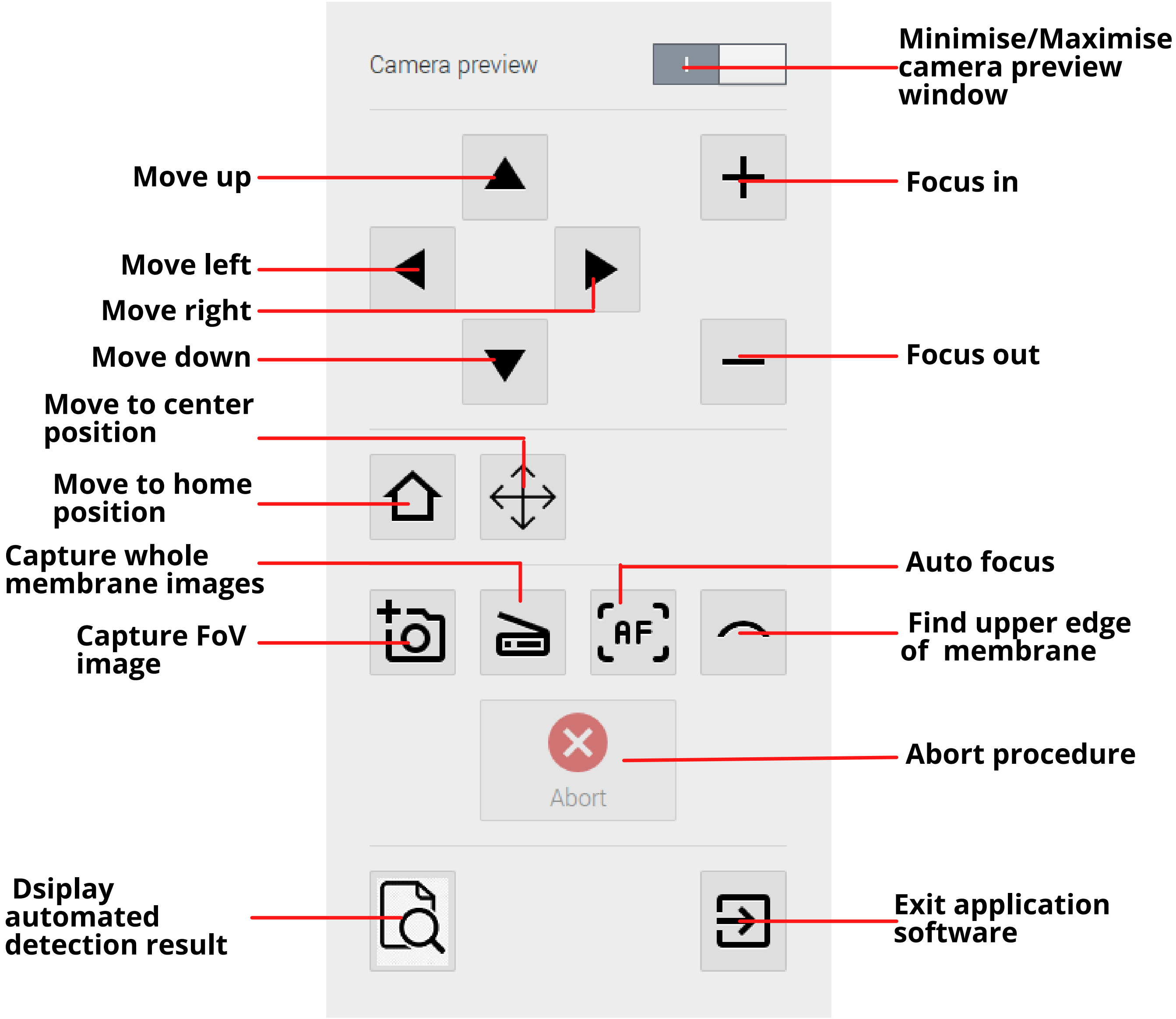

Keyboard

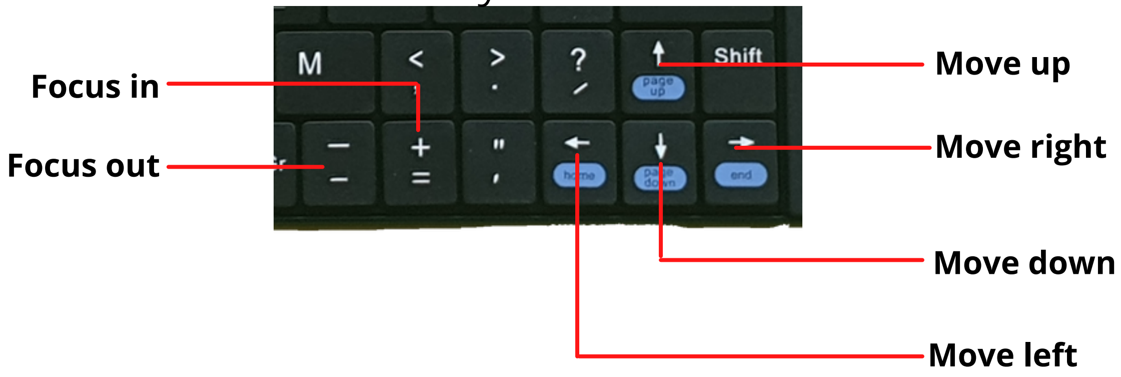

Start-up Schistoscope

1.Power on the Schistoscope using the power switch located at the back of the device. Booting takes about 15 seconds.

2.Launch Schistoscope Graphical User Interface by double clicking the Schistoscope dekstop icon

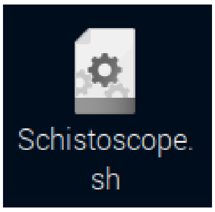

3.Select "Execute in Terminal" on the pop-up window

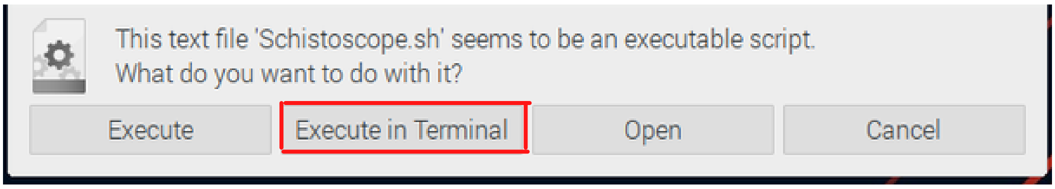

Capture Whole Membrane Images

1.Insert sample slide into the slide holder compartment as shown on the image below

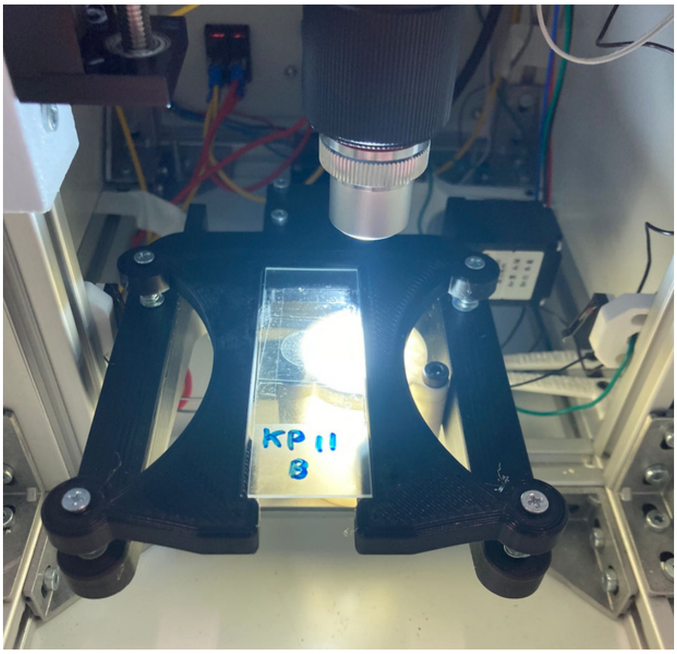

2. Adjust the objective such that it's placed directly on the membrane using the left, right, up and down arrow buttons.

3. Focus on the sample using the "+" and "-" button

Capture Whole Membrane Images...

3. Move sample stage using the "Move up/down/left/right" buttons until the **topmost** edge of the filter membrane is in field of view displayed on the device screen as shown below

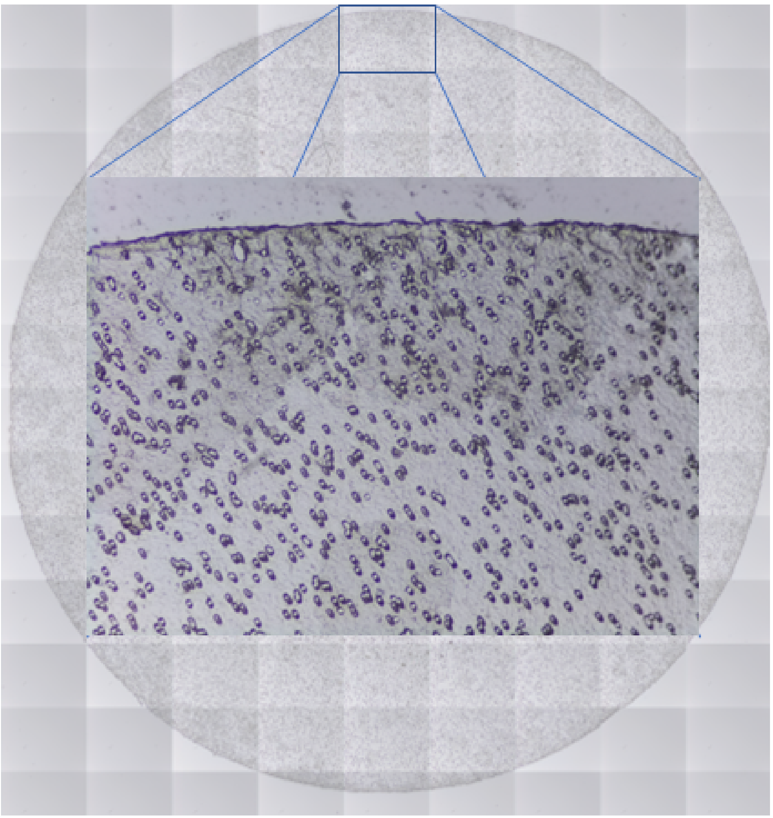

4. Click 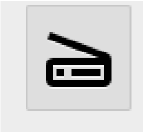 button to capture images of the whole filter membrane.

Capture Whole Membrane Images

5. A pop-up window will appear. Enter the sample ID **and press the "enter" keyboard key**. Select the membrane size 25mm on the drop down menu. leave the other parameters unchanged

6. To start the scanning procedure, click "Begin" button.

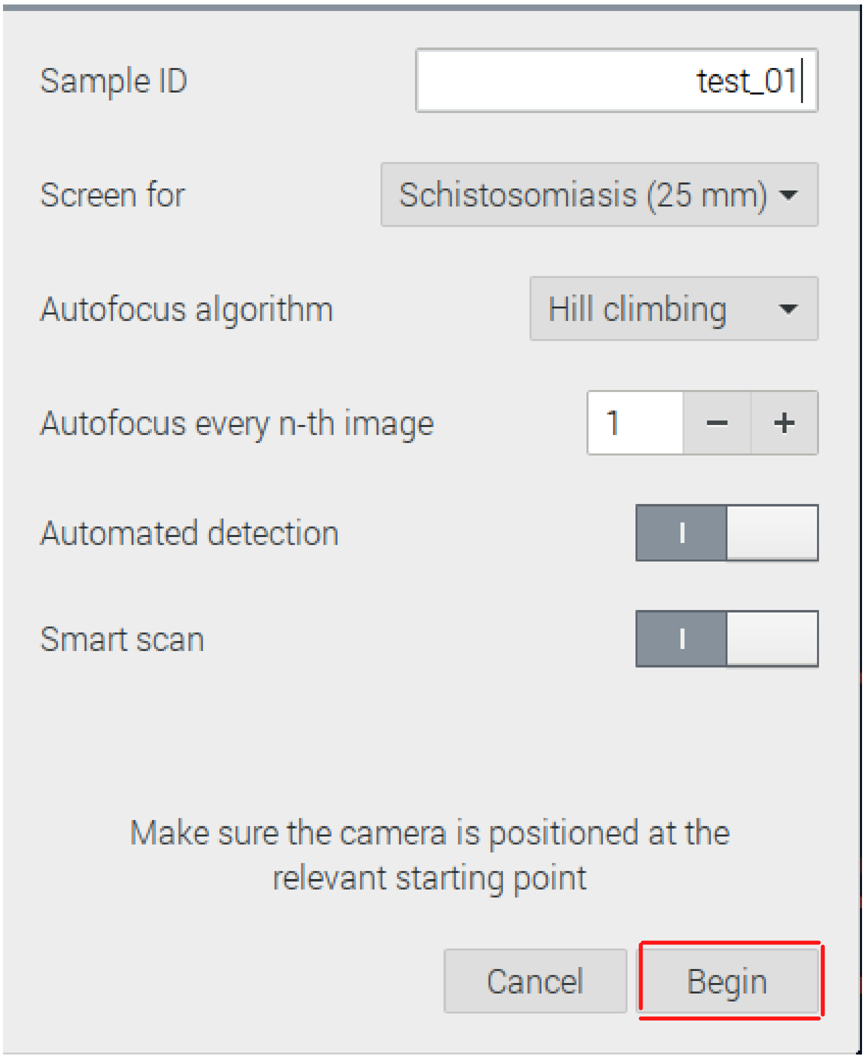

Automated Egg Detection

7. At the end of the scan, the AI result window pops-up. This window can also be displayed by clicking the button 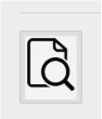

8. In AI result window, confirm the sample ID and that, the number of images scanned is 372. Then record the egg count on the sheet provided.

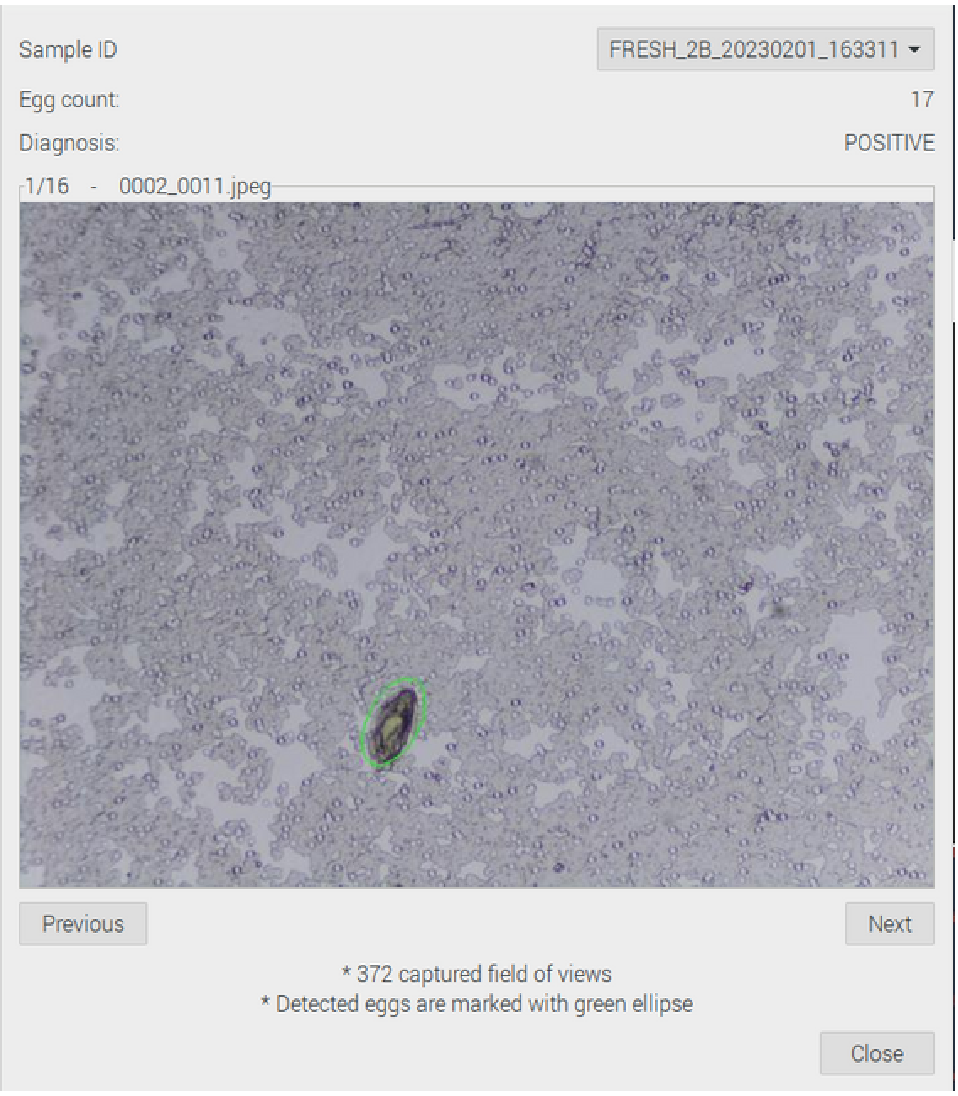

9. Captured sample images folders can be found in the directory "home\Schistoscope Data"

10. Click the 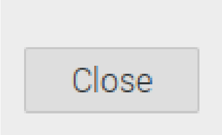

button to exit the result output window and repeat the process from step 1 of whole membrane capture to scan another sample
