# Supplementary material for: Validation of artificial intelligence-based digital microscopy for automated detection of Schistosoma haematobium eggs in urine in Gabon
Source: PLoS Negl Trop Dis. 2024 Feb 23;18(2):e0011967. doi: 10.1371/journal.pntd.0011967 (PMC10917302; doi:10.1371/journal.pntd.0011967)
Supplement: Attachment — Submitted filename: Response to reviewers comments.docx [file pntd.0011967.s009.docx]

**Response to Reviewer’s Comment**

Journal   : PLoS Negl Trop Dis
Reference : PNTD-D-23-01306

Dear Editor,

We thank you and the reviewers for the critical feedback and constructive suggestions which have allowed us to improve the content and clarity of the manuscript. Please find below a point-by-point response to the specific comments. Attached is a revised version of the manuscript with track-changes, as well as a clean version.

Our responses are italicized and colored blue. The line numbers in the responses refer to the revised manuscript with track-changes.

**Editor’s feedback**

In this study Meulah and colleagues optimise a quantitative methodology to accurately diagnose schistosomiasis. Even though the study is timely and within the scope of the journal, several issues raised by the three reviewers and the editor need first to be thoroughly addressed before considering it suitable for publication.

•	The Title is probably too long, it could be shorter and punchier. Please consider something along these lines:

“Artificial intelligence-based digital microscopy for automated detection of Schistosoma haematobium eggs in urine.”

Thank you for your recommendation. *The title has been shortened “**Validation of artificial intelligence-based digital microscopy for automated detection of Schistosoma haematobium eggs in urine in Gabon” (L1-2).*

•	Line 139: “The Schistoscope was assumed to have a sensitivity and specificity non-inferior to conventional microscopy, which were realistically assumed to be 80% and 90%, respectively based on field expert estimates using real-time PCR and UCP-LF CAA.” Provide a reference to back up the sensitivity and specificity of conventional microscopy.

*Thank you for pointing out this observation. We are aware of several studies exploring day-to-day variation of S. haematobium egg-count and other studies with indications on the diagnostic sensitivity of conventional microscopy [1-3]. However, to our knowledge there are no publications dedicated to diagnostic performance of conventional microscopy especially with this set of composite reference. One of the main issues here is that a proper reference test to compare conventional microscopy to is lacking [4]. In addition, microscopy, being a highly operator dependent procedure, sensitivity and specificity in general should only be interpreted as rough indications.*

•	Line 247: Please, provide reference to support the selection criteria for levels of infection intensity.

*Infection intensities criteria was done based on World Health Organisation categorisation and the low infection intensity was further split to determine the performance of the Schistoscope at very low infection intensities. The reference to support infection intensity category has been added (L255).*

•	Results headings – consider writing headings that are more descriptive and highlight the main/ key findings described in the section

*The results sub-headings have been modified with more details “diagnostic performance of the Schistoscope on freshly prepared samples” (L266) and “diagnostic performance of the Schistoscope on banked samples (L309)” and the main findings highlighted L267-299 and L310-341 respectively.*

•	PCR; is the positive control the same as the internal control? What kind of normalization is used during the qPCR analysis (ratio unknown: control ? What were the Cq values of the NTC? Did the authors run a standard curve along with controls?). All these technical points are extremely important not only because this study focuses on describing a new diagnostic methodology, but for the sake of transparency and reproducibility.

*Thank you for your thoughtful and detailed suggestions. We appreciate your attention to the technical aspects of our study and acknowledge the importance of transparency and reproducibility of the method.*

*The positive control is different from the internal control. The positive control used contained S. haematobium template DNA (Ct-value 24±1, determined with standards) included as a duplicate per PCR plate. The internal control was included in every sample prior to DNA extraction to address sources of variability and ensure reliability of our results. Normalisation during PCR analysis was done based on the Ct-value of the internal control* *(28±2). Also, samples with Ct-values for the internal control out of this range were repeated (From DNA extraction to amplification). The non-template control (NTC) had no Ct-­­value and if so the whole plate was termed invalid and rerun. More details about the assay have been added at (L210-229).*

**Reviewer's Responses to Questions**

Key Review Criteria Required for Acceptance?> As you describe the new analyses required for acceptance, please consider the following:<

**Methods**

-Are the objectives of the study clearly articulated with a clear testable hypothesis stated? -Is the study design appropriate to address the stated objectives? -Is the population clearly described and appropriate for the hypothesis being tested? -Is the sample size sufficient to ensure adequate power to address the hypothesis being tested? -Were correct statistical analysis used to support conclusions? -Are there concerns about ethical or regulatory requirements being met?

Reviewer #1: Yes

Reviewer #2: Minor additions of detail are needed

•	The legend for figure 1 could be more descriptive

*The legend to Figure 1 has been made more descriptive “comprehensive flow chart detailing the methodical sequence of urine sample collection, processing by the Schistoscope, conventional microscopy, real-time PCR and UCP-LF CAA and data analysis” (L131).*

•	Line 152 – you say Whatman membranes were used to capture the eggs but how was this done e.g. using a syringe and filter holder ?

*The sentence has been adjusted accordingly (L157).*

•	Line 213-224 – some more detail is needed

o	What is DEC

*DEC is DNA extraction control. DEC is already defined in L210. Slight adjustments have been made to make it clear (L211-229).*

o	What was the internal control amounts used (DNA, primers and probe)

*DEC 670 KIT is supplied by the manufacturer in 2 vials. One with the internal control DNA and the other a mixture of primers and probes. 5μL of internal control DNA was added to each sample prior to DNA extraction (L210-211) and 0.4μL of the control mix was added to the PCR reaction mixture (L220).*

o	What was the Schisto positive control

*Schistosoma positive controls are samples previously identified as positive for S. haematobium infection with Ct-values 24±1 determined with standards.*

o	Why was 40 cycles used as the cut off

*The 40 cycles was set as a cut-off based on the minor modifications of the reaction mixture and volume used in Obeng et al 2008 as described in L214-217 and was validated in CERMEL.*

o	What was the CT value of the internal control

*The Ct-value of the internal was 28±2.*

o	How has the internal control been validated as a multiplex with the ITS primers?

*Validation of the internal control as well as ITS-2 targets were done in a singleplex and in a multiplex as previously described [5-7].*

•	Line 243 – what is CRS ? write in full before using the acronym

*CRS is composite reference standard and was already written in full (L115).*

Reviewer #3: The objectives of the study are clearly articulated with a clear testable hypothesis, and correct statistical analysis were used to support conclusions stated. However, the study design is not quite clear. To me, it is not necessary to have the B1 and B2 components; Just having fresh samples and banked samples should be enough to avoid unnecessary complexity. The B1 component can be deleted.

*Thank you for your comment. Because of operational limitations, we unfortunately did not have the option to test all urine samples with all four tests. Especially in the set of banked samples (the B-study) we could not perform both real-time PCR and the UCP-LF CAA test in about 60% of the urines. Not only do we think all collected data should in principle be reported, we also find it relevant to demonstrate that the association between the Schistoscope and conventional microscopy is consistent for all subsets.*

Also, the sample size calculation seems not appropriate, it seems like the formula for a cross sectional survey was used for sample size calculations. I didn’t attempt to calculate the sample size but I do think that a different formula integrating the minimal difference expected between both tests (Schistoscope vs microscopy and Schistoscope vs DNA- or Antigen-based assays) should be used (please see Page 10, Lines 245-247 for the hypothesis).

*Thank you for your comment. For this study a cross-sectional sampling was carried out. However, based on our hypothesis (Schistoscope non-inferior to microscopy), the formula for comparing diagnostics for a match-paired design and non-inferiority was used to determine the number of samples enough to estimate a less than 10% difference in the diagnostic sensitivity and specificity between methods [8]. To determine non-inferiority of the Schistoscope to microscopy, both methods were evaluated with the same and more accurate reference standard, i.e. a composite of UCP-LF CAA and real-time PCR, with the same sample power. The focus on microscopy for non-inferiority is because it is the most commonly used method in endemic settings for control of schistosomiasis.*

Regarding the ethical regulatory requirements (please see Page 7, Lines 135-137), this is not a clinical trial, and I am wondering why this study was registered on clinicaltrials.gov. If this study just took advantage of an existing study, it is worth to mention it. Also, the authors declared that they provided treatments to all infected individuals. Knowing that the test and treat procedures are sometimes complicated, especially when the test cannot be completed in a few minutes, I am curious to know how the authors managed to find and treat all those positive patients. This can be helpful for other studies.

*Thank you for your comments. CERMEL, being a centre for clinical trials, it is mandatory for any study interacting directing or indirectly and making use of human samples, to be registered at clinicaltrials.gov. Also, as explained in the manuscript this study did not only explore ongoing studies but also performed an independent cross-sectional sampling as stated in L121. Indeed the test and treatment procedure for all infected persons can be complicated. However, CERMEL has a well-structured system for handling this. Sampling is performed during the week, while results and treatments are provided during the weekends. Secondly, we had in place field workers (community contacts) to facilitate communication with the community leaders and participants. Also, the communities are rather small, making it easier to find participants and treat them with praziquantel on an individual base when schistosomiasis has been diagnosed.*

**Results**

-Does the analysis presented match the analysis plan?-Are the results clearly and completely presented?-Are the figures (Tables, Images) of sufficient quality for clarity?

Reviewer #1: Yes

Reviewer #2: A minor addition to the results would be beneficial

•	Did you find other discrepancies that are challenging to interpret – eg egg positive but CAA neg and DNA neg.

*Yes we found a number of cases that were egg positive by the Schistoscope but negative by real-time PCR and UCP-LF CAA. Also for conventional microscopy these discrepancies were noted. These findings were already reported in L289-291 and L324-327 for study A and study B, respectively. The challenges concerning specificity were discussed in L368-L372.*

Reviewer #3: The results are clearly presented and match the analysis plan. However, one of the main assets of this technology/device is the ability to be used for the diagnosis of schistosomiasis on banked samples. However, the design developed by the authors do not fully enable to better capture that asset.

Indeed, it would have been interesting to have the results of conventional microscopy for all the samples, not only for a subset of samples used as quality control. Even if quality control should be considered, I am quite curious to have the results of the quality control to be able to figure out to what extent was the variation between the two readings, and whether it was unidirectional or random. This should be clearly presented and discussed.

Also, the issue of artefact raised at page 20, Lines 350-358 can be important for conventional microscopy as well. As such, it would have been worth to analyze all the banked slides, or present and discuss the results of quality control.

*Thank you for your comments. A subset of the banked samples was randomly selected and re-analysed by conventional microscopy to check for the viability and quality of the biobank. The Wilcoxon sign rank test was used to compare the egg count before storage with the egg count after storage, which revealed a non-significant difference. Because the outcome did not suggest a deterioration of the samples during the storage period, we didn’t find it necessary to perform re-analysis of all biobanked samples. Furthermore, the distribution of egg counts before and after storage was similar. The data set will be made available online in a data repository.*

*Regarding the issue of artifacts, we manually checked several images from a set of samples, showing discrepancies between the Schistoscope and conventional microscopy and noted that the fresh samples had comparatively more components that could mistakenly be interpreted as eggs (i.e. artefacts) than the banked samples. We think this has more to do with the fact they have been collected in a different time period and in a slightly different community, than with the storage conditions. This has been discussed in L360-372.*

Page 9, Lines 191-193. It is unclear how the discrepancies between conventional microscopy readings between the two readers was done? Was a third reader involved as adjudicator or a third reading was done by the same two microscopists? This should be stated clearly.

*Thank you for your question. The statement has been adjusted and we assume it is more clear now(L197).*

**Conclusions**

-Are the conclusions supported by the data presented? -Are the limitations of analysis clearly described? -Do the authors discuss how these data can be helpful to advance our understanding of the topic under study?-Is public health relevance addressed?

Reviewer #1: Yes

Reviewer #2: The conclusions are well supported and the limitations are clearly described. I think the thoughts around the egg number cutoff to categorise intensity rather than actual egg counts is a good way forward.

Other minor comment are:

•	Line 381 – could the difference also relate to the fact you did not bead beat the eggs so the DNA may not have been released ?

*Thank you for your question. Indeed, bead beating prior to DNA extraction has been shown to improve the DNA yield and subsequently leads to reduction of Ct-values. However, this reduction does not, by itself, improve the correlation between egg counts and Ct-values [9].*

•	Mention the potential for screening stool?

*Thank you for your suggestion. We have so far only validated the Schistoscope performance on urine analysis. And we find it a very strong statement to conclude about its potential on stool without proper validation on stool. However, we have added a line to explore the Schistoscope potential for screening stool sample in the discussion (L428).*

Reviewer #3: The conclusions are globally supported by the data presented. However, there are a number of issues that need to be addressed:

First and foremost, the main conclusion or finding of the manuscript is not convincing enough. In fact, the authors found that Schistoscope is non inferior to conventional microscopy which is already a poor diagnostic test especially when it comes to talk about transmission interruption or elimination. It is well known and widely accepted that when the parasitic loads are low, the sensitivity of microscopy is low and more sensitive tools are therefore needed. This should be raised as a limitation of the study/manuscript. I would like to acknowledge that Schistoscope has a good potential, and the tool can still be improved. Indeed, I had the opportunity to review the manuscript presenting the early versions of the device which was clearly inferior to microscopy. The current version of the device, even if non-inferior to conventional microscopy, is significantly less performant than DNA- and antigen-based assays (please see figures at page 16), and it might be worth to clearly indicate the potential applications of Schistoscope.

It is unclear to me how the authors try to justify the difference between Schistoscope and PCR or CAA (Page 20, Lines 372-375). There is not just a matter of target but sensitivity and specificity of the techniques. This should be clearly explained.

*Thank you for your comment. The main aim of developing the Schistoscope is to support the schistosomiasis control strategies and to advance towards elimination as a public health problem, as clearly stated in L78-85, and not to achieve elimination by transmission interruption. And given that microscopy is still the most widely used method in endemic settings for control strategies, the Schistoscope could be a matching solution if it performs as well as conventional microscopy, mainly because of the fact that it is an automated procedure.*

*Indeed such a test will be insufficient in settings approaching transmission interruption. There, alternatives like the UCP-LF CAA and real-time PCR, will be required. Furthermore, the Schistoscope, just as conventional microscopy, being an egg based detection method, is limited in sensitivity for the low infection intensities, as observed and discussed in this study (L356-358). A few references have been added (L352) regarding potential applications of such a tool. In addition, the decrease in correlation between the Schistoscope and real-time PCR/UCP-LF CAA as a measure of parasite quantification has been explained (L377-388). For reference, the differences in PCR and CAA patterns has been extensively discussed in Hoekstra et al., [10]. In that study, post-praziquantel treatment data suggest a relevant difference between egg-based and worm-based diagnostic findings. Furthermore, differences in diagnostic sensitivity and specificity between methods was shown to have more impact on the proportion of positives and not so much on parasite quantification.*

The authors mentioned the limitations (time of Schistoscope operation) of the study (Page 22, Lines 420-433) that raised another concern or issue. It would have been worth to compare the time of operations of Schistoscope to that of conventional microscopy in the same circumstances. This is important and aligns with the applications of Schistoscope mentioned above.

*Thank you for your comment. We do agree with you on the importance of time of operation of the Schistoscope in comparison to that of conventional microscopy. However, this was not within the scope of this study. Furthermore, as extensively discussed, the time of operation could be reduced to less than 10 mins when applied on different filters (L433-438). An evaluation of the time of operation is to be undertaken following further optimisation and customisation of the current version of the Schistoscope.*

Editorial and Data Presentation Modifications?- Use this section for editorial suggestions as well as relatively minor modifications of existing data that would enhance clarity. If the only modifications needed are minor and/or editorial, you may wish to recommend “Minor Revision” or “Accept”.

Reviewer #1: Minor Revision

Reviewer #2: (No Response)

Reviewer #3: This manuscript needs some language editing or correction of typos. For example, please consider “as previously described” not “described previously” (page 9 line 209); “internal transcribed spacer” not “internally transcribed spacer” (page 9 line 210); “ct” not “cq” (page 9 line 233) …

*These corrections have been effected and throughout the manuscript Cq has been changed to Ct-value.*

Authors should avoid some of abbreviations or explain them at their first used. For example “UCP-LF-CAA” and “CRS” in the abstract …

*We thank the reviewer for pointing this out and adaptations have been made to explain these abbreviations (L31-33).*

Some of the references used in the manuscript are not appropriately presented. For example, WHO should be numbered as all other references …

*This has been sorted (L76-83 and L438).*

**Summary and General Comments**

Use this section to provide overall comments, discuss strengths/weaknesses of the study, novelty, significance, general execution and scholarship. You may also include additional comments for the author, including concerns about dual publication, research ethics, or publication ethics. If requesting major revision, please articulate the new experiments that are needed.

Reviewer #1:

General Comment

This manuscript is well written and well-structured and the study was well designed. I enjoyed reading! Please, only few comment for improvement.

Abstract

**= “**CRS**”** used for the first time should be write in full

*CRS has been written in full (L31) as “composite reference standard”.*

Introduction

**=** L72 : “Out of approximately 250 million affected people worldwide” No more true ! Recent estimation is around 150 million. Please, revise including a reference

*We thank the reviewer for pointing out this. Although we could not find your quoted estimate, the phrase has been edited and properly referenced (L76-79).*

Methods

= L123-L125 : “Microscopy slides were subsequently biobanked for retrospective analysis with the Schistoscope (~2 years later).” What do you mean by “biobanked”? For reproducibility purpose, more details need to be provided here. Where? How many times after preparation? At which temperature?

*From urine collected from each participant, a microscopy slide was prepared as described in L156-161. After being read by conventional microscopy, the slides have been stored at* *4 °C in appropriate boxes. More details are to be found in L156-161.*

Discussion

The authors could trust to add few sentences first, on the fact that the performance of the Schistoscope should be also assessed in dry setting mostly with very high temperature such as Niger and Tchad and where schistosomiasis is prevalent. Second, some cost-effectiveness analysis are warranted support the use of the current Schistoscope in large scale intervention.

*We thank the reviewer for the suggestions. A small paragraph has been added describing the need for further evaluation under different climate conditions and the importance of a cost-effectiveness study (L425-L430).*

Reviewer #2: This study fully evaluates the sensitivity and specificity of the Schistoscope for eggs detection in urine. This is a large and detailed study that warrants publication. The methods are sound and the data interpretation has been well executed. I look forward to further developments of the Schistoscope as its limitations are addressed.

*Thank you for your thoughtful and positive review of our study evaluating the sensitivity and specificity of the Schistoscope for S. haematobium egg detection in urine. We are pleased you found the evaluation comprehensive and well-executed.* *Addressing the device limitations is one of the major next steps in taking this prototype to the next phase and contributing more to this field.*

Reviewer #3: The manuscript submitted by Meulah and colleagues entitled “Extended laboratory validation of the performance of an artificial intelligence-based digital microscope (Schistoscope) in Lambaréné (Gabon) for automated detection of Schistosoma haematobium eggs in urine” aimed to validate the performance of a novel AI-based diagnostic tool, the Schistoscope, for the detection and quantification of Schistosoma haematobium eggs in urine using conventional microscopy and a composite diagnosis (real-time PCR and a lateral flow assay) as a reference. Although the accurate diagnosis of schistosomiasis is of high interest and an up-to-date issue in the framework of transmission interruption/elimination (in its 2021-2030 roadmap, one of the critical actions recommended by the WHO is the development of diagnostic tests, including standardized point-of-care diagnostic), the paper lacks clarity, especially in its design.

*Thank you for your insightful feedback on our manuscript. We appreciate your recognition of the importance of accurate schistosomiasis diagnosis in the context of schistosomiasis control and advancement to elimination. We hope that based on the responses provided to your comments and questions regarding the design of our study (see above) clarifies obscurity about our design.*

References

1.	Coulibaly JT, Andrews JR, Lo NC, N'Goran EK, Utzinger J, Keiser J, et al. Schistosoma haematobium Egg Excretion does not Increase after Exercise: Implications for Diagnostic Testing. Am J Trop Med Hyg. 2018;98(3):772-5. Epub 20171214. doi: 10.4269/ajtmh.17-0728. PubMed PMID: 29260647; PubMed Central PMCID: PMCPMC5930907.

2.	Knopp S, Corstjens PL, Koukounari A, Cercamondi CI, Ame SM, Ali SM, et al. Sensitivity and Specificity of a Urine Circulating Anodic Antigen Test for the Diagnosis of Schistosoma haematobium in Low Endemic Settings. PLoS Negl Trop Dis. 2015;9(5):e0003752. Epub 20150514. doi: 10.1371/journal.pntd.0003752. PubMed PMID: 25973845; PubMed Central PMCID: PMCPMC4431728.

3.	Van Etten L, Kremsner PG, Krijger FW, Deelder AM. Day-to-day variation of egg output and schistosome circulating antigens in urine of Schistosoma haematobium-infected school children from Gabon and follow-up after chemotherapy. Am J Trop Med Hyg. 1997;57(3):337-41. doi: 10.4269/ajtmh.1997.57.337. PubMed PMID: 9311646.

4.	Beltrame A, Guerriero M, Angheben A, Gobbi F, Requena-Mendez A, Zammarchi L, et al. Accuracy of parasitological and immunological tests for the screening of human schistosomiasis in immigrants and refugees from African countries: An approach with Latent Class Analysis. PLoS Negl Trop Dis. 2017;11(6):e0005593. Epub 20170605. doi: 10.1371/journal.pntd.0005593. PubMed PMID: 28582412; PubMed Central PMCID: PMCPMC5472324.

5.	Verweij JJ. Application of PCR-based methods for diagnosis of intestinal parasitic infections in the clinical laboratory. Parasitology. 2014;141(14):1863-72. Epub 20140429. doi: 10.1017/s0031182014000419. PubMed PMID: 24780241.

6.	Verweij JJ. Validation and maintaining laboratory developed molecular tests compliant with ISO15189 for diagnosis of intestinal parasitic infections. Expert Rev Mol Diagn. 2022;22(6):595-601. Epub 20210902. doi: 10.1080/14737159.2021.1971974. PubMed PMID: 34424112.

7.	Verweij JJ, Stensvold CR. Molecular testing for clinical diagnosis and epidemiological investigations of intestinal parasitic infections. Clin Microbiol Rev. 2014;27(2):371-418. doi: 10.1128/cmr.00122-13. PubMed PMID: 24696439; PubMed Central PMCID: PMCPMC3993103.

8.	Machin D, Campbell MJ, Tan SB, Tan SH. Sample size tables for clinical studies: John Wiley & Sons; 2011.

9.	Pomari E, Perandin F, La Marca G, Bisoffi Z. Improved detection of DNA Schistosoma haematobium from eggs extracted by bead beating in urine. Parasitol Res. 2019;118(2):683-6. Epub 20181112. doi: 10.1007/s00436-018-6137-7. PubMed PMID: 30417247; PubMed Central PMCID: PMCPMC6349810.

10.	Hoekstra PT, Casacuberta-Partal M, van Lieshout L, Corstjens PLAM, Tsonaka R, Assaré RK, et al. Limited efficacy of repeated praziquantel treatment in Schistosoma mansoni infections as revealed by highly accurate diagnostics, PCR and UCP-LF CAA (RePST trial). PLOS Neglected Tropical Diseases. 2022;16(12):e0011008. doi: 10.1371/journal.pntd.0011008.
