# Supplementary material for: Validation of artificial intelligence-based digital microscopy for automated detection of Schistosoma haematobium eggs in urine in Gabon
Source: PLoS Negl Trop Dis. 2024 Feb 23;18(2):e0011967. doi: 10.1371/journal.pntd.0011967 (PMC10917302; doi:10.1371/journal.pntd.0011967)
Supplement: Attachment — Submitted filename: Reviewer Comment (30 11 2023).docx [file pntd.0011967.s008.docx]

Author   : Meulahet al.
Title       : Extended laboratory validation of the performance of an artificial intelligence-based digital microscope

(Schistoscope) in Lambaréné (Gabon) for automated detection of *Schistosoma haematobium* eggs in urine
Journal   : PLoS Negl Trop Dis
Reference : PNTD-D-23-01306
    

**General Comment**

This manuscript is well written and well structured and the study was well designed. I enjoyed reading! Please, only few comment for improvement.

**Abstract**

**= “**CRS**”** used for the first time should be write in full

**INTRODUCTION**

**=** L72 : “Out of approximately 250 million affected people worldwide” No more true ! Recent estimation is around 150 million. Please, revise including a reference

**METHODS**

= L123-L125 : “Microscopy slides were subsequently biobanked for retrospective analysis with the Schistoscope (~2 years later).” What do you mean by “biobanked”? For reproducibility purpose, more details need to be provided here. Where? How many times after preparation? At which temperature?

DISCUSSION

The authors could trust to add few sentences first, on the fact that the performance of the Schistoscope should be also assessed in dry setting mostly with very high temperature such as Niger and Tchad and where schistosomiasis is prevalent. Second, some cost-effectiveness analysis are warranted support the use of the current Schistoscope in large scale intervention.
